# Risk factors associated with *Streptococcus pneumonia* carriage in children under five years old with acute respiratory infection in Niger

**DOI:** 10.11604/pamj.2019.33.239.15945

**Published:** 2019-07-19

**Authors:** Ibrahim Dan Dano, Sani Ousmane, Kamaye Moumouni, Adamou Lagare, Idi Issa, Jean Testa

**Affiliations:** 1 Centre de Recherche Médicale et Sanitaire (CERMES), 634 Boulevard de la Nation, Niamey, Niger; 2 Hôpital National de Niamey (HNN), Service de Pédiatrie A, Niamey, Niger

**Keywords:** Risk factors, nasopharyngeal carriage, S. pneumonia, children under five years

## Abstract

**Introduction:**

*Streptococcus pneumonia* is a leading cause of bacterial pneumonia, meningitis and sepsis in children, and pneumococcal carriage is an important source of horizontal spread of these pathogens within the community.

**Methods:**

A questionnaire was addressed to parents for the collection of sociodemographic and medical information. Nasopharyngeal swabbing was processed using a molecular method. We used logistic regression models to examine independent associations between pneumococcal carriage and potential risk factors. All associations with a p-value of < 0.25 in the bivariate regression analyses were subsequently entered in the multivariate regression model.

**Results:**

A total of 637 children aged 1 to 59 months admitted for acute respiratory infection were included. The rate of respiratory virus carriage was 76%, whereas that of bacteria was 47% and that of bacteria-virus co-colonization was 42%. A bivariate analysis showed that carriage was not related to gender, father's or mother's education level, father's occupation, type of housing or lighting, or passive exposure to cigarette smoking in the house. It was also not linked to complete vaccination with PCV-13 or PPSV-23 and antibiotic treatment prior to hospitalization. A multivariate analysis showed that carriage was related to age greater than 3 months, maternal occupation, house flooring type, and co-colonization of another bacterium and virus.

**Conclusion:**

These results can be helpful to understand the dynamics of pneumococcal nasopharyngeal colonization; they confirm the interest of vaccinating infants before the age of 3 months with appropriate vaccine to prevent spread nasopharyngeal colonization and pneumococcal diseases in children.

## Introduction

*streptococcus pneumonia* is a significant human pathogen and a leading cause of bacterial pneumonia, meningitis, and sepsis in children [1]. *S. pneumonia* causes an estimated 11% (8-12%) of all deaths in children aged 1-59 months [1]. *S. pneumonia* is a commensal of the human respiratory tract that is carried by many people without becoming ill. An important feature is that pneumococcal diseases will not occur without preceding nasopharyngeal (NP) colonization with a homologous strain [2]. Cross-sectional studies have shown that, at any given time, approximately 20 - 50% of healthy children harbour at least one serotype of this bacterium in the rhinopharynx, whereas longitudinal studies have shown that almost all children can carry this bacterial species [3, 4]. Pneumococcal carriage is an important source of horizontal spread of these pathogens within the community [5]. Such pneumococcal NP carriage studies are important because carriage has a key role in pneumococcal disease and pneumococcal spread. We assessed risk factors associated with the carriage of *S. pneumonia* in children under five with signs of respiratory infection.

## Methods

**Study design:** this cross-sectional study was conducted in the paediatric departments of the National Hospital of Niamey and that of Lamordé (Niger). Children aged 1 to 59 months with signs of respiratory infection (severe cough or tachypnea with axillary temperature above 38°C) who were hospitalized in one of the paediatric departments of the two hospitals were enrolled from January 2015 to June 2016. Children under the age of one month or over the age of five years and children suffering from heart disease or chronic respiratory infection were excluded. NP samples were obtained for molecular identification. Data on potential risk factors were gathered by confidential interview based on a questionnaire.

**Ethical considerations:** this study was approved by the National Consultative Ethics Committee of Niger by decision No 0016/2013/CCNE on 30 October 2013; approved consent from the parents of the children was requested before inclusion.

**Nasopharyngeal swabbing:** nasopharyngeal swabbing was performed with the nasopharyngeal flocked swabs kit, UTM^TM^ 350C, Copan Diagnostics. The swabs were inserted along the nasal septum just above the floor of the passage to the nasopharynx until resistance was met; the swab was rotated gently against the NP mucosa for 10 - 15 seconds and then gently removed. After the swab removed from the patient, it was placed into the tube of UTM^TM^ transport medium all the way to the bottom of the tube.

**Serum procalcitonin (PCT) level dosage:** the serum PCT level was determined for the diagnosis of bacterial infection with the BRAHMS PCT kit (reference 30450) on a VIDAS machine (BioMérieux SA, France). A PCT level below 0.1 μg/L was considered normal, 0.1 to 2 μg/L was considered moderate and above 2 μg/L was considered high.

**RT- PCR for the detection of viral and bacterial NP colonization:** NP carriage of *S. pneumonia* was determined by a multiplex real-time PCR with the FTD Respiratory pathogens 21 plus (Ref: FTD-2+.1-32. Fast-Track Diagnostics Luxembourg Sarl). This test enables the detection of the following respiratory pathogens: influenza A (Flu A), influenza A (H1N1) swl, influenza B (Flu B); coronaviruses NL63 (Cor63), 229E (Cor229), OC43 (Cor43) and HKU1 (CorHKU1); parainfluenza 1, 2, 3 and 4 (Para1, Para2, Para3, Para4); human metapneumovirus A and B (HMPVA and B); rhinovirus (Rhino); respiratory syncytial viruses A and B (RSV A and B); adenovirus (AV); enterovirus (EV); parechovirus (PV); bocavirus (HboV); *Mycoplasma pneumonia* (Mpneu); *Chlamydia pneumonia* (Cpneu); *Streptococcus pneumonia* (Spneu); *Haemophilus influenza* B (HIB); and *Staphylococcus aureus* (Saur).

**Statistical analysis:** statistical analyses were performed with Epi info 7. We used logistic regression models to examine independent associations between pneumococcal carriage and potential risk factors. All associations with a p-value of < 0.25 in the bivariate regression analyses were subsequently entered in the multivariate regression model. Odd ratios and adjusted odd ratios with their 95% confidence intervals were computed. P-values ≤ 0.05 were considered statistically significant.

## Results

**Figure 1:**
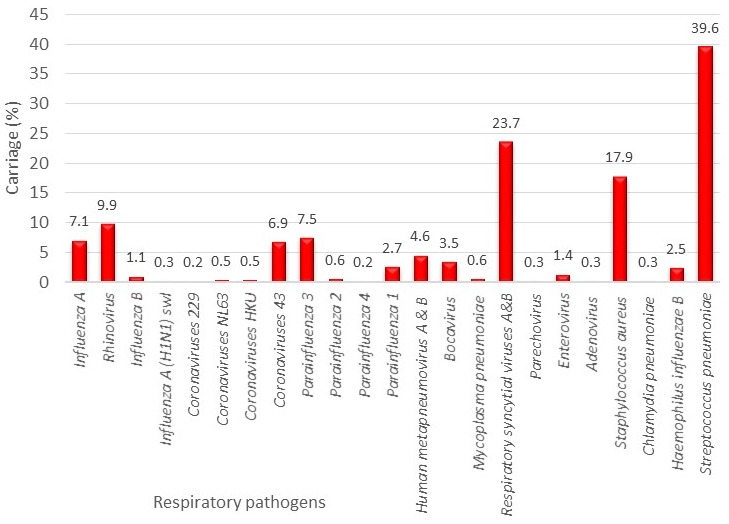
Preceding nasopharyngeal bacterial and viral carriage

**Figure 2:**
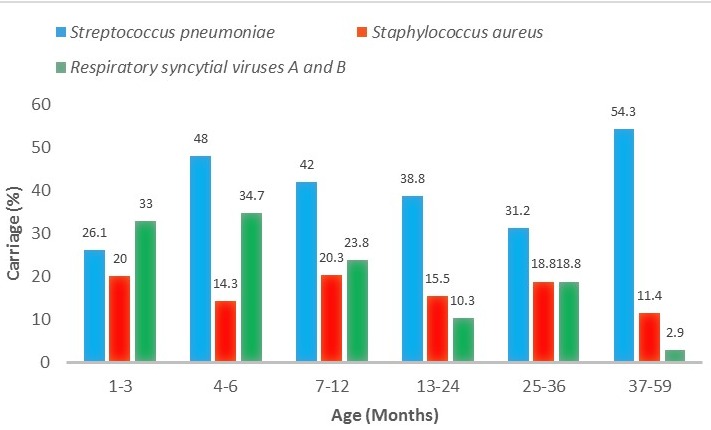
NP carriage of main pathogens detected according to age

A total of 637 children aged 1 to 59 months admitted for acute respiratory infection were included in this study. The mean age of the children was 13.2 ± 12.65 months, and the female to male ratio was 0.8. The rate of respiratory virus carriage was 76%, that of bacteria carriage was 47% and that of bacteria-virus co-colonization was 42%. The most detected bacteria was *S. pneumonia* (39.6%), and the most detected virus was respiratory syncytial viruses A and B (23.7%), followed by rhinovirus (9.9%) and influenza A (7.1%). Details of the NP carriage rate of the respiratory pathogens detected are summarized in Figure 1, and the distribution according to age of the main pathogens detected is shown in Figure 2. In the bivariate analysis, age older than 3 months (except for 24-36 months), house flooring material of tile, mother's profession of civil servant, non-attendance at a day care centre and co-carriage with another bacteria or virus were risk factors for *S. pneumonia* carriage (Table 1,Table 2). No association was found with regard to sex, duration of breast feeding, educational level, father's profession, type of house and electric lightning type of house, number of children less than 15 years old in the family, vaccination status, infection and prior antibiotic treatment. Risk factors for *S. pneumonia* carriage in the multivariate logistic analysis were age older than 3 months (except for 24-36 months), house flooring material of tile, mother's profession of civil servant and co-carriage with another bacteria or virus (Table 3). In the simple logistic regression analysis, attendance at a day care centre was inversely related to *S. pneumonia*carriage, but the association was not significant in the multivariate model.

**Table 1:** Sociodemographic potential risk factors estimated by bivariate logistic regression for the carriage of *S. pneumonia* in children under five years old with acute respiratory infection

Characteristic		Number of children	Carriage *S. pneumoniae* (%)	OR	95% CI	*P*
Age (months)	1 - 3	128	28.1	1		
	4 - 6	121	49.6	2.51	1.48 – 4.24	< 0.001
	6 - 12	161	41.0	1.77	1.08 – 2.91	< 0.05
	12 - 24	136	41.2	1.86	1.06 – 2.99	< 0.05
	24 - 36	53	30.2	1.10	0.54 – 2.22	NS
	>36	37	45.9	2.17	1.02 – 4.61	< 0.05
Sex	Female	290	38.3	1		
	Male	347	40.6	1.10	0.80 – 1.51	NS
Mother’s occupation	Trader	20	25.0	1		
	Civil servant	114	50.8	3.10	1.05 – 9.11	< 0.05
	House wife	393	38.2	1.83	0.65 – 5.16	NS
	Other	76	35.5	1.65	0.54 – 5.04	NS
Father’s occupation	Other	196	34.8	1		
	Civil servant	135	43.1	1.35	0.87 – 2.15	NS
	Trader	210	40.0	1.21	0.81 – 1.82	NS
	Farmer	70	38.0	1.11	0.65 – 1.92	NS
Mother’s educational level	Primary	139	33.8	1		
	Secondary	183	41.5	1.39	0.87 – 2.19	NS
	University	42	50.0	1.95	0.97 – 3.93	NS
	Islamic study	114	39.5	1.24	0.74 – 2.09	NS
	None	112	41.1	1.36	0.81 – 2.28	NS
Father’s educational level	None	91	38.5	1		
	Primary	95	40.0	1.09	0.60 – 1.98	NS
	Secondary	194	36.6	0.95	0.56 – 1.59	NS
	University	70	44.3	1.30	0.69 – 2.47	NS
	Islamic study	155	39.3	1.06	0.62 – 1.82	NS
Type of housing	Cement built house	308	39.6	1		
	Other	313	38.5	1.1	0.74-1.5	NS
House floor material	Cement	317	34.7	1		
	Tile	44	59.1	2.74	1.43 – 5.21	< 0.01
	Ground	250	40.8	1.30	0.92 – 1.84	NS
Electric lightning	No	179	38.55	1		
	Yes	444	39.9	1.05	0.74 – 1.50	NS
Number of children less than 15 years old	1 - 2	199	39.2	1		
	3 - 4	209	38.8	0.98	0.65 – 1.46	NS
	≥ 5	159	39.6	1.01	0.66 – 1.55	NS
Attendance at a day care centre	Yes	139	30.2	1		
	No	497	42.5	1.67	1.12 – 2.51	< 0.05
Passive exposure cigarette smoke	Yes	111	44.4	1		
	No	387	36.7	0.73	0.47 – 1.12	NS

Abbreviation: OR, odds ratio; CI, confidence interval; NS, not significant

**Table 2:** Medical potential risk factors estimated by bivariate logistic regression for the carriage of *S. pneumonia* in children under five years old with acute respiratory infection

Characteristic		Number of children	Carriage of *S. pneumoniae*(%)	OR	95% CI	*p*
Co-carriage with another bacteria	Yes	133	60.9	1		
	No	504	33.9	2.99	2.01 – 4.44	< 10^-4^
Co-carriage with a virus	Yes	488	46.7	1		
	No	149	16.1	4.54	2.83 – 7.28	< 10^-4^
Co-carriage with another bacteria and virus	yes	125	62.4	1		
	No	512	34.0	3.18	2.11 – 4.77	< 10^-4^
Vaccination against pneumococci	Yes	54	42.5	1		
	No	583	39.2	1.14	0.65 – 2.01	NS
Vaccinated with PCV-13	Yes	16	43.7	1		
	No	621	39.4	1.19	0.43 – 3.24	NS
Vaccinated with PPSV-23	Yes	40	42.5	1		
	No	597	39.3	1.13	0.59 – 2.17	NS
Prior antibiotic treatment	Yes	135	36.3	1		
	No	301	42.2	0.78	0.51 – 1.18	NS
Breast feeding duration	≤ 6 months	169	45,0	1		
	6 - 12 months	97	43,3	0.93	0.56 - 1.54	NS
	> 12 months	114	43,0	0.92	0.57 - 1.49	NS
Procalcitonin level (bacterial infection)	≤ 0.1 μg/L	129	43.4	1		
	0.1 – 2 μg/L	286	37.4	0.77	0.51 – 1.18	NS
	> 2 μg/L	160	39.9	0.88	0.52 – 1.35	NS

Abbreviation: OR, odds ratio; CI, confidence interval; NS, not significant

**Table 3:** Adjusted ORs estimated by multivariate logistic regression for *S. pneumoniae* carriage, according to possible risk factors

Risk factor		aOR	95% CI	*P*
Age (months)	1 - 3	1		
	4 - 6	2.42	1.33 – 4.42	< 0.01
	6 - 12	1.97	1.12 – 3.48	< 0.05
	12 - 24	1.86	1.03 – 3.34	< 0.05
	24 - 36	1.11	0.51 – 2.43	NS
	> 36	2.42	1.04 – 5.60	< 0.05
Mother’s occupation	Trader	1		
	Civil servant	3.1	1.05 – 9.11	< 0.05
	House wife	1.83	0.65 – 5.16	NS
	Other	1.65	0.54 – 5.04	NS
*S. pneumo*niae co-carriage with another bacteria	Yes	1		
	No	2.71	1.74 – 4.23	< 10^-4^
*S. pneumoniae* co-carriage with a virus	Yes	1		
	No	4.06	2.39 – 6.91	< 10^-4^
Attendance at a day care centre	Yes	1		
	No	0,79	0,50-1,27	NS
Flooring of the house	Cement	1		
	Tile	2.87	1.40 – 5.89	< 0.01
	Ground	1.30	0.92 – 1.84	NS

Abbreviation: aOR, adjusted odds ratio; CI, confidence interval; NS, not significant

## Discussion

The nasopharynx is known to be the main ecological reservoir of *S. pneumonia* and is where the bacteria give rise to disease after extending to other areas of the respiratory tract or penetrating normally sterile body fluids [6]. Although NP isolates are not useful for predicting the causative agent of invasive disease in individuals, they reflect epidemiological aspects of pneumococcal disease in the community [7]. In developing countries, particularly in Niger, there are few studies describing the epidemiology of NP carriage of *S. pneumonia*. This study focused on children under five years old with signs of respiratory infection. Most NP pneumococcal carriage studies enrolled children [8] or younger children [9-12]. Several studies have included healthy children [13] whereas others included children with concomitant respiratory infection [11]. This study described the epidemiology of NP carriage of *S. pneumonia*. The bacterial carriage rate obtained in this study was comparable to those in previous studies in younger children [3, 4]; likewise, the viral carriage rate was also comparable [14-16]. Carriage of *S. pneumonia* was associated with age; the peak incidence of carriage in children is observed during the first 3 years of life [2], and the lowest carriage rate is observed in infants younger than 2 months of age [17]. The mean age of first acquisition of *S. pneumonia* was reported to be 6 months with a range of 1-30 months [18-20]. Therefore, age is an important risk factor for pneumococcal colonization, and its influence varies with the phase of growth [21]. A positive association between the carriage of other pathogens and the NP carriage of *S. pneumonia* was found in this study. Bacterial carriage is known to be influenced by bacterial and viral intra-and inter-species interactions (commensal and pathogenic) [22]. These interactions can be synergistic or antagonistic. In general, the risk of *S. pneumonia* seemed to increase in the presence of particular respiratory viruses [14]. The carriage of *S. aureus* has been reported to be inversely related to *S. pneumonia* carriage [23] likely due to bacterial interference [14]; however, this study failed to show such a negative association. It is unknown whether this association is direct or dependent on other determinants [14] such as age [23] and season [24]. Environmental features are risks factors for *S. pneumonia* carriage [17]. Low socio-economic status was also reported as a risk factor for the colonization and carriage of respiratory pathogens, likely due to cramped and poor housing [22].

The mother's occupation appeared to affect *S. pneumonia* carriage more than the father's profession in this study. Pneumococcal carriage was reported to be associated with exposure to other children [19, 22, 24]. Frequent and close person-to-person contact favours the development and transmission of pneumococci. Attendance at a day care centre was reported to be a risk factor for pneumococcal carriage [22], particularly in developed countries [25]. In this study, attendance at a day care centre was surprisingly inversely associated with pneumococcal carriage in the bivariate analysis, but it was not significant in the multivariate analysis. This could be a consequence of a confounding effect. The high prevalence of carriage in developing countries is a possible explanation of the lack of association between pneumococcal carriage and attendance at a day care centre [26]. Close contact and poor hygienic conditions cannot explain increased pneumococcal carriage in day care centres. Additional factors are apparently associated, such as age, number of children attending the day care centre and pathogen interference. No association between pneumococcal carriage and the number of children less than 15 years old in the family was found. A study in Gambia [12] also failed to identify an association with the number of children in the house or children sleeping in the same room. The relationship between the carriage of *S. pneumonia* and educational level is not clear. Although some studies showed a close relationship between low educational level of the parents and higher nasopharyngeal colonization [12], other studies [13, 27, 28] such this study failed to show this association. Although reported in some studies [12, 29-31], this study did not identify any associations between the carriage of *S. pneumonia*, smoking habits, breast feeding and recent antibiotic use. However, the results obtained showed that when the house floor material was tile, the prevalence of carriage increased. A study performed in Gambia [26] did not identify this association, but they did not consider tile in their design. Several studies from different parts of the world [31-33] have demonstrated the ability of different pneumococcal conjugate vaccines to reduce the rate of pneumococcal carriage. It has been reported [34] that after vaccination with a conjugate vaccine, rapid replacement of pneumococci by new serotypes not included in the vaccine occurs; thus, cross protection can also occur. This might explain the lack of association between pneumococcal carriage and vaccination with PCV-13 observed in this study, which did not identify the different pneumococcal serotypes. Unlike other studies [14,35, 36], no association between NP carriage of *S. pneumonia* and antibiotic pre-treatment was found, as reported by another study [37]. The differences with other studies suggest that the factors that influence colonization are multiple and not entirely clear [22]. This study included children with signs of respiratory infection who were visiting a paediatric care centre; thus, these results may not represent a healthy paediatric population.

## Conclusion

These results can be helpful to understand the dynamics of pneumococcal nasopharyngeal colonization; they confirm the interest of vaccinating infants before the age of 3 months with appropriate vaccine to prevent nasopharyngeal colonization and pneumococcal diseases in children.

### What is known about this topic

- Pneumococcal nasopharyngeal colonization is generally high in children;
- The rate of pneumococcal nasopharyngeal colonization vary by region;
- Pneumococcal nasopharyngeal colonization were affected by many factors.

### What this study adds

- Description of risks factors associated to pneumococcal nasopharyngeal colonization in Niger;
- Risks factors associated to pneumococcal nasopharyngeal colonization in children observed in Niger differ from those observed in developed countries;
- Age appear as the most common risk factor observed and is important for preventive measures.

## Competing interests

The authors declare no competing interest.
